# Muscle stem cell polarity requires QKI-mediated alternative splicing of Integrin Alpha-7 (Itga7)

**DOI:** 10.26508/lsa.202101192

**Published:** 2022-02-14

**Authors:** Claudia Dominici, Stéphane Richard

**Affiliations:** Segal Cancer Center, Lady Davis Institute for Medical Research and Gerald Bronfman Department of Oncology and Departments of Medicine, Human Genetics and Biochemistry, McGill University, Montréal, Québec, Canada

## Abstract

Muscle stem cells (MuSCs) have the ability to carry out the specialized function of cell polarization, which is required for the production of one repopulating cell and one myogenic progenitor cell with muscle regeneration capabilities. The mechanisms which regulate proteins involved in establishing MuSC polarity such as Dmd and Itga7 are currently not well understood. Herein, we define the RNA-binding protein Quaking (QKI) as a major regulator alternative splicing of key MuSC polarity factors including Dmd, Itga7, Mark2, and Numb. We generate a conditional QKI knockout mouse, and for the first time it is shown in vivo that deficiency of QKI in MuSCs results in reduced asymmetric cell divisions, leading to a loss of the myogenic progenitor cell population and striking muscle regeneration defects. Transcriptomic analysis of QKI-deficient MuSCs identifies QKI as a regulator of the splicing events which give rise to the mutually exclusive Itga7-X1 and -X2 isoforms. We observe increased X1 expression in QKI-deficient MuSCs and recapitulate this splicing event using antisense oligonucleotide directed against a quaking binding site within the Itga7 mRNA. Interestingly, recreating this single splicing event is detrimental to the polarization of Itga7 and Dmd proteins, and leads to a drastic reduction of the myogenic progenitor population, highlighting the significance of QKI-mediated alternative splicing of Itga7 in maintaining MuSC polarity. Altogether, these findings define a novel role for QKI as a post-transcriptional regulator of MuSC polarity.

## Abstract (precis)

The RNA-binding protein Quaking (QKI) is a post-transcriptional regulator of genes encoding polarity proteins in muscle stem cells. Loss of QKI in MuSCs results in reduced myogenic progenitors and a striking muscle regeneration defect.

## Introduction

Muscle regeneration is a complex process which begins with the activation of tissue-resident quiescent muscle stem cells (MuSCs) (Montarras et al, 2013). After muscle injury or disease, activated MuSCs may divide symmetrically to replenish the MuSC pool, or asymmetrically to give rise to one repopulating stem cell and one myogenic progenitor cell (Kuang et al, 2007). Lineage progression of myogenic progenitor cells ends with terminal differentiation and fusion to construct new muscle fibers, thus completing muscle regeneration (Chargé & Rudnicki, 2004; Le Grand & Rudnicki, 2007; Relaix & Zammit, 2012; Gurevich et al, 2016). The specific subcellular localization of certain proteins is a requirement for asymmetric MuSC division. For example, dystrophin (Dmd)-deficient MuSCs were observed to have asymmetric division defects as a result of faulty localization of polarity-determining proteins Mark2 and Pard3, leading to a loss of myogenic progenitor cells (Dumont et al, 2015). More recently, it has been shown that treatment of MuSCs with exogenous EGF leads to polarized activation of the EGF receptor-Aurora kinase A signaling pathway which then regulates asymmetric MuSC division (Wang et al, 2019). In addition, the Numb protein segregates to one daughter cell during mitosis, along with an unequal distribution of template DNA to provide different identities to the resulting daughter cells (Shinin et al, 2006). These findings lay important groundwork towards a better understanding of the complexities of asymmetric MuSC division. However, the role of post-transcriptional regulatory networks mediated by RNA-binding proteins (RBPs) in cell polarity and asymmetric MuSC division is not understood.

The QKI RBP belongs to the hnRNP K homology (KH)–type family of RBPs (Ebersole et al, 1996; Lukong et al, 2008). QKI is a specific RBP recognizing the following QKI response element (QRE): ACUAAY (1–20) UAAY (Y; C/U) (Galarneau & Richard, 2005) as a dimer (Chen & Richard, 1998; Beuck et al, 2012; Teplova et al, 2013). QKI is known to regulate RNA metabolism (Darbelli & Richard, 2016), in part, by influencing pre-mRNA splicing (Wu et al, 2002). There are three main isoforms named QKI-5, QKI-6, and QKI-7 for the length of their mRNAs which encode identical proteins except for the last 35 amino acids of their C terminus (Ebersole et al, 1996; Darbelli & Richard, 2016). QKI-5 contains a C-terminal nuclear localization signal, whereas QKI-6 and QKI-7 do not (Pilotte et al, 2001). QKI-deficient cells have defects in alternative splicing (AS) networks, mainly attributed to the lack of the nuclear QKI-5 isoform (Hall et al, 2013; van der Veer et al, 2013; Zong et al, 2014; de Bruin et al, 2016; Lee et al, 2020; Chen et al, 2021).

The role of the QKI proteins and AS, in general, in MuSC physiology is not well understood. In the present study, we report that deletion of QKI in mouse MuSCs leads to reduced asymmetric MuSC divisions, causing a deficit in myogenic progenitors and severe muscle regeneration defects after injury. Transcriptomic analysis identified aberrant splicing of known regulators of MuSC asymmetric division, including *Itga7*, *Dmd*, *Mark2*, and *Numb*. We show that QKI-deficient MuSCs were unable to polarize the location of Itga7 and Dmd proteins within the cell before division. Interestingly, using a 2′-O-methyl antisense oligonucleotide (ASO) to mask the QRE within *Itga7* intron 4 near the 3′ splice site of exon 5 was sufficient to induce loss of polarized localization of Dmd and Itga7 proteins, as observed in QKI-deficient MuSCs. These findings define QKI as a regulator of *Itga7* AS, which is required for MuSC polarity and the production of myogenic progenitors after MuSC activation.

## Results

### QKI-5 is expressed during the activation of primary skeletal MuSCs and throughout myogenesis

**Figure 1.**
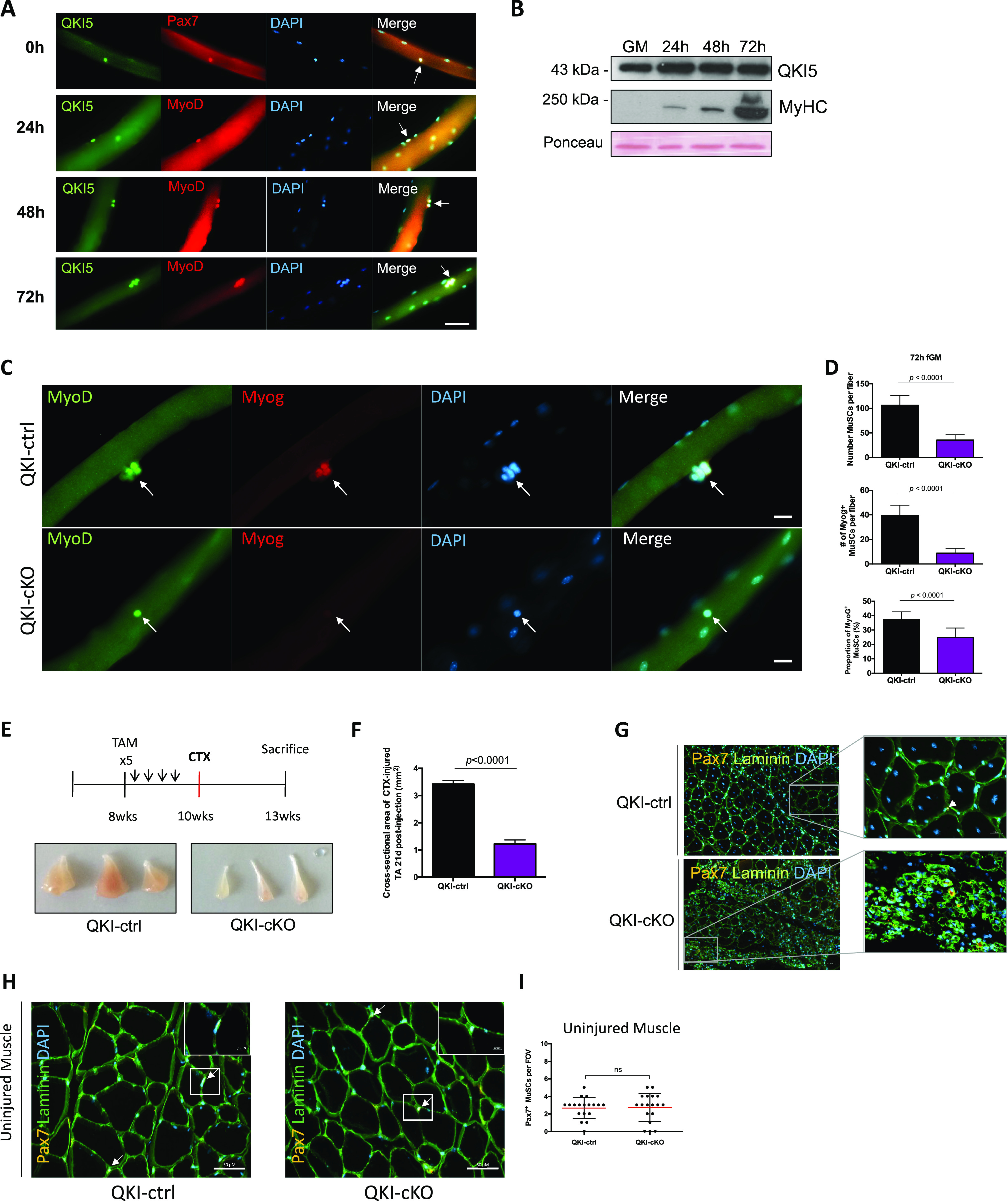
Mice with QKI-depleted muscle stem cells (MuSCs) exhibit reduced myogenic progenitors and defects in skeletal muscle regeneration. **(A)** Representative immunofluorescence images of myofibers isolated from wild type mice and immunostained for QKI-5 (green), and co-stained with appropriate MuSC markers (Pax7 and MyoD; Red), counterstained with DAPI. Fibers were fixed immediately after isolation (0 h, quiescent MuSCs), and after 24, 48, and 72 h of culture. White arrows denote MuSCs. Scale white bar represents 50 μm. **(B)** Western blot of QKI-5 protein expression during a differentiation time course of primary mouse MuSCs (GM denotes growth media; 24–72 h represent time in differentiation media; MyHC is myosin heavy chain). Ponceau red was used to show equal protein loading. **(C)** Muscle fibers isolated from QKI-ctrl and QKI-cKO mice and cultured in fiber growth media for 72 h, stained with MyoD (green), Myogenin (red), and counterstained with DAPI (blue) and merged. Scale bar represents 10 μm. **(C, D)** Quantification of Myogenin-expressing MuSCs (upper panel) and total MuSCs (middle panel) from (C) (n = 3 biological replicates, minimum 1,000 cells quantified per condition, *P* < 0.0001, unpaired *t* test). Proportion of Myog + MuSCs from (C, lower panel) (n = 3 biological replicates, minimum 1,000 cells quantified per condition, *P* < 0.0001, unpaired *t* test). **(E)** Timeline of 4-hydroxytamoxifen injections (once daily for 5 d) to induce conditional QKI knockout in MuSCs of QKI^2lox/2lox^:Pax7^CreERT2/+^ or QKI^2lox/2lox^:Pax7^+/+^ as ctrl, followed by cardiotoxin injection in the tibialis anterior (TA) hindlimb muscle to induce muscle injury. 3 wk after injury, the mice were sacrificed and their TA muscles isolated (n = 6 biological replicates, three replicates depicted in bottom panels). **(E, F)** Quantification of cross-sectional area in square millimeter of TA muscles from QKI-ctrl and QKI-cKO mice in (E). **(G)** Representative immunofluorescence cross-sectional images of TA muscles 3 wk after cardiotoxin injury from QKI-ctrl and QKI-cKO. Laminin (green) stains muscle fiber edges, Pax7 (orange) indicates MuSCs, counterstained with DAPI (blue) (n = 6). **(H)** Cross-section of uninjured contralateral TA muscle of QKI-ctrl and QKI-cKO mice, laminin (green), Pax7 (orange), and DAPI (blue). Pax7^+^ MuSCs magnified in insets. Scale bars represent 50 μm. **(H, I)** Quantification of Pax7^+^ MuSCs per field of view in QKI-ctrl and QKI-cKO TA cross sections represented in (H).

To define the role of QKI-5–mediated AS during the early stages of activation when MuSCs exit quiescence and begin to proliferate, we confirmed its expression in myofiber-associated MuSCs. The first MuSC division on an ex vivo cultured myofiber occurs at ∼40 h after isolation and by 72 h the MuSCs have proliferated extensively (Dumont et al, 2015). To monitor QKI-5 expression at these critical times, we isolated myofibers from C57BL/6 mice and cultured them for 0 (quiescent), 24, 48, or 72 h. Myofibers were co-immunostained with anti-QKI-5 antibody and antibodies against MuSC markers Pax7 (at 0 h) or MyoD (at 24, 48, and 72 h) to identify MuSCs. We observed that nuclear QKI-5 was expressed at all time points (Fig 1A), indicating its presence throughout the activation and proliferation phases of MuSCs in their niche during ex vivo myofiber culture.

We next interrogated QKI-5 expression throughout myogenesis of primary MuSCs. Primary MuSCs were purified from C57BL/6 mice and differentiated in vitro with reduced serum media for 24, 48, and 72 h. Total cell lysates were immunoblotted with antibodies against QKI-5 and the terminal differentiation marker myosin heavy chain to confirm that MuSCs were differentiated. QKI-5 protein was increased immediately after 24 h of differentiation and persisted through 72 h (Fig 1B). Together, these data show that the expression of QKI-5 increases during MuSC differentiation.

### Mice with QKI-depleted MuSCs exhibit reduced myogenic progenitors and defects in skeletal muscle regeneration

**Figure S1.**
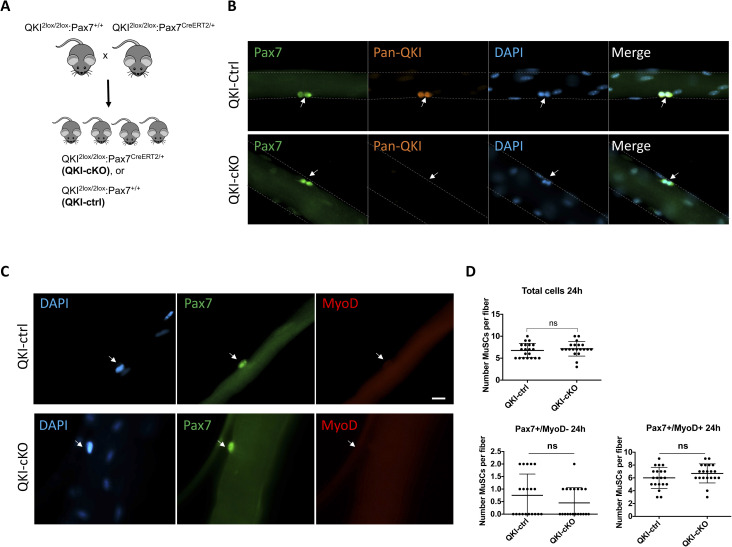
Mice with QKI-depleted MuSCs exhibit reduced myogenic progenitors and defects in skeletal muscle regeneration. **(A)** The breeding strategy for QKI-cKO and QKI-ctrl mice is illustrated. **(B)** Myofibers isolated from QKI-ctrl and QKI-cKO mice were cultured for 48 h in fiber growth media and were immunostained with Pax7 (green) and pan-QKI (orange) antibodies, and counterstained with DAPI (blue). Muscle stem cells (MuSCs) are indicated with white arrows. **(C)** Myofibers isolated from QKI-ctrl and QKI-cKO mice and cultured for 24 h in fiber growth media were assessed for Pax7 (green) and MyoD (red) expression. MuSCs are indicated with white arrows. Scale bar represents 10 μm. **(C, D)** Quantification of total MuSCs from (C) (upper panel), Pax7+/MyoD− and Pax7+/MyoD+ (lower panels) (n = 3 biological replicates, minimum 100 cells per group was quantified, ns, not significant).

Because QKI-5 is expressed during MuSC activation and differentiation, we examined whether its presence was necessary for these processes. Conditional QKI knockout mice were generated by crossing QKI^2lox/2lox^ mice with Pax7^CreERT2/+^ mice (Murphy et al, 2011) to deplete QKI in MuSCs (QKI^2lox/2lox^;Pax7^CreERT2/+^, herein referred to as QKI-cKO, Fig S1A). QKI^2lox/2lox^; Pax7^+/+^ littermates were used as controls, herein referred to as QKI-ctrl. Daily injections of 4-hydroxytamoxifen (TAM) for 5 d were performed on QKI-ctrl and QKI-cKO mice, and myofibers were isolated and cultured ex vivo for 72 h revealing QKI ablation in MuSCs by immunostaining with a pan-QKI antibody (Fig S1B). Interestingly, the total number of MuSCs per myofiber at 72 h was reduced in QKI-cKO (35.5 ± 10.7) compared with QKI-ctrl mice (106.3 ± 18.5) (Fig 1C and D). In contrast, the number of MuSCs was not significantly altered shortly after activation at 24 h of culture with 6.8 ± 0.4 in QKI-ctrl versus 7.2 ± 0.4 in QKI-cKO mice, nor was Pax7/MyoD expression status (Fig S1C and D). We then quantified the number of differentiating myogenic progenitors based on immunostaining of the differentiation marker, myogenin (Myog). We observed a drastic reduction in the number of Myog^+^ MuSCs per QKI-cKO myofibers (8.8 ± 4.0) compared with QKI-ctrl (39.4 ± 8.5) (Fig 1C and D). These findings indicate that QKI deficiency does not negatively affect viability or early stages of MuSC activation before the first cell division but has a marked effect on the expansion of myogenic progenitors on ex vivo cultured myofibers.

We reasoned that the reduced number of myogenic progenitors in QKI-cKO mice might influence muscle regeneration after cardiotoxin (CTX) injury of the tibialis anterior (TA) hindlimb muscle. Therefore, we injected CTX into the TA hindlimbs of QKI-ctrl and QKI-cKO mice (Fig 1E). 3 wk after injection, QKI-cKO mice had a remarkably reduced TA size with a cross-sectional area of 1.2 mm^2^ ± 0.07 in QKI-cKO versus 3.4 mm^2^ ± 0.06 in QKI-ctrl (Fig 1E and F), and disorganized muscle fiber architecture (Fig 1G). Notably, the uninjured contralateral TA muscle which also contained QKI-deficient MuSCs did not have significantly different Pax7^+^ MuSC number (2.7/field of view ± 0.3 in QKI-ctrl, 2.7/field of view ± 0.4 in QKI-cKO) or muscle fiber architecture (Fig 1H and I), consistent with our observation of unchanged MuSC number in cultured myofibers at 24 h (Fig S1D). Together, these data suggest that QKI is required for the generation of myogenin-expressing myogenic progenitors and muscle regeneration after injury.

### QKI-deficient primary skeletal MuSCs drastically down-regulate markers of terminal differentiation

**Figure 2.**
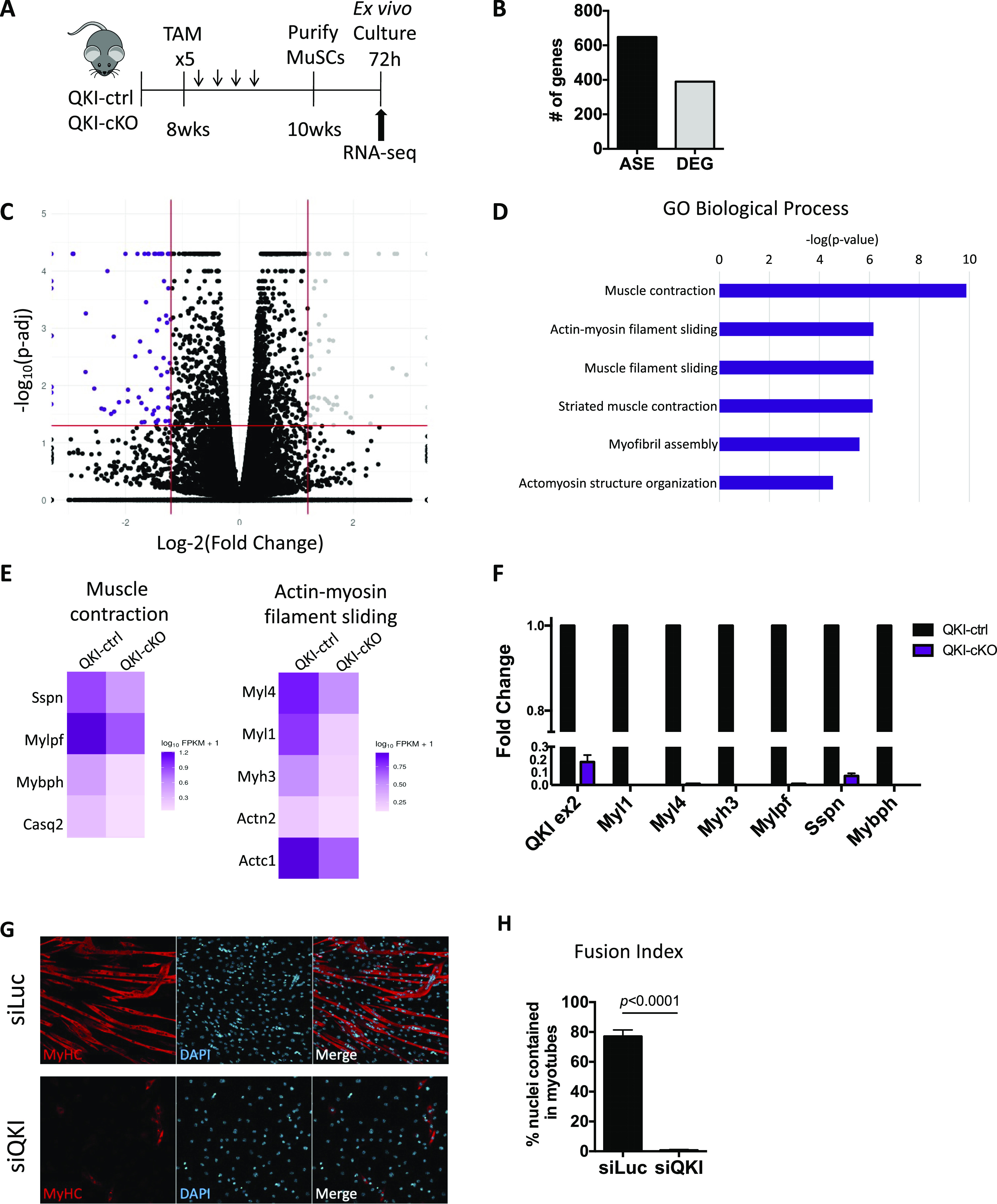
QKI-deficient primary skeletal muscle stem cells (MuSCs) drastically down-regulate markers of terminal differentiation. **(A)** Timeline for 4-hydroxytamoxifen-induced knockout of QKI in MuSCs, MuSC purification and culture before RNA isolation for RNA-seq (n = 3 biological replicates). **(B)** Total alternative splicing events and differentially expressed genes in QKI-cKO MuSCs compared with QKI-ctrl. **(C)** Volcano plot showing differentially regulated transcripts of QKI-ctrl and QKI-cKO MuSCs. Vertical lines represent log-twofold change cutoff of value 1.2, and horizontal lines represent false discovery rate cutoff value 0.05. **(D)** Pathway enrichment analysis of GO biological processes carried out using Enrichr software of significantly down-regulated genes in QKI-cKO versus QKI-ctrl MuSCs. **(E)** Heat maps show the down-regulation of genes selected from enriched gene sets corresponding to *muscle contraction* and *actin-myosin filament sliding* terms. Colour scale represents log_10_(fpkm) + 1 values. **(E, F)** Quantitative PCR confirmation of selected down-regulated myogenic differentiation markers in QKI-cKO MuSCs compared to QKI-ctrl from (E), and exon 2 of QKI transcript to confirm knockdown (error bars represent mean ± SEM, n = 3 biological replicates). **(G)** Immunofluorescence of C2C12 myotubes differentiated for 72 h in the presence of siQKI or siLuc control. Myosin heavy chain is in red and the slides were counterstained with DAPI (blue). **(G, H)** Fusion index of myotubes from (G) calculated as percentage of nuclei within myotubes (error bars represent mean ± SEM, n = 3 independent experiments, *P* < 0.0001).

**Figure S2.**
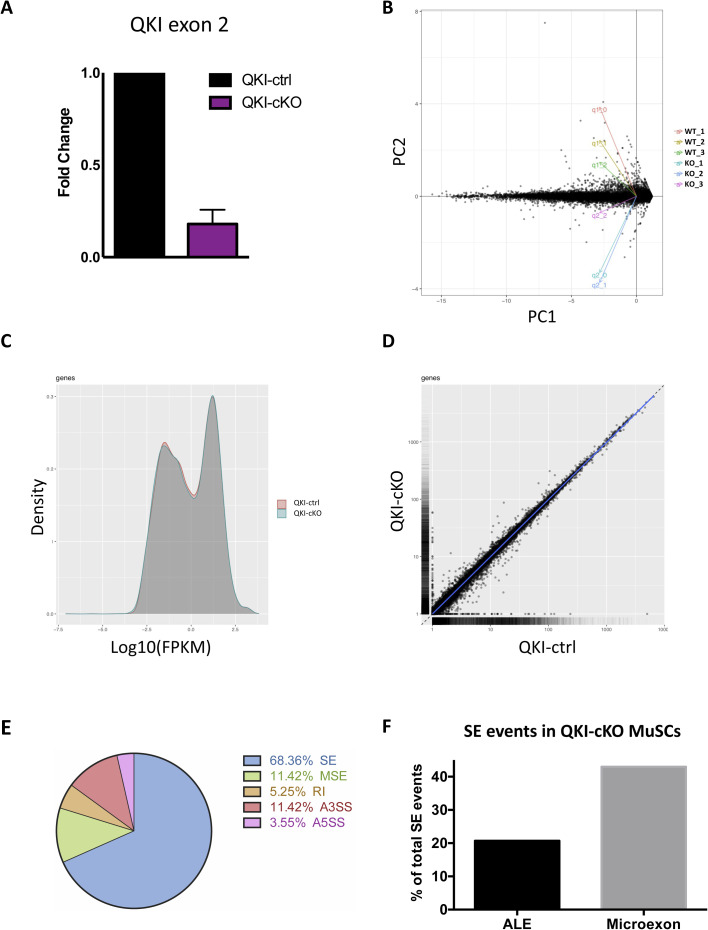
QKI-deficient primary skeletal MuSCs drastically down-regulate markers of terminal differentiation. **(A)** RT-qPCR confirmation of QKI knockout in muscle stem cells used for RNA-seq (n = 3 biological replicates normalized to Gapdh levels, *P* = 0.0065 unpaired *t* test). **(B)** PCA plot of individual replicates. Dimensionality reduction was performed with cummeRbund software using Cufflinks output. **(C)** Distribution of FPKM scores across QKI-cKO and QKI-ctrl conditions from one replicate performed using cummeRbund software. **(D)** Pairwise scatterplot of gene expression from QKI-cKO and QKI-ctrl conditions. **(E)** Pie chart representing the summary of alternative splicing events in QKI-cKO muscle stem cells compared to QKI-ctrl (SE, skipped exon; MSE, multiple skipped exons; RI, retained intron; A3SS, alternative 3′ splice site; A5SS, alternative 5′ splice site). **(F)** Percentage of SE events which contained alternative last exon or microexons.

To determine if the AS events were regulated by QKI in MuSCs, we injected TAM daily for 5 d into QKI-ctrl and QKI-cKO mice, and purified MuSCs for ex vivo expansion. After 72 h in culture, total RNA was isolated and paired-end RNA sequencing was performed (n = 3 biological replicates) (Fig 2A). CRE-mediated deletion of *QKI* exon 2 was confirmed with RT-qPCR (Fig S2A). Differentially expressed genes (DEGs) were revealed after analysis with Cufflinks v2.2.1 (Trapnell et al, 2010) and AS analysis was carried out using rMATS v4.0.2 software (Shen et al, 2014). A PCA plot was generated after dimensionality reduction performed using the CummeRbund software package (Trapnell et al, 2012) (Fig S2B), and gene expression was visualized with distribution of fragments per kilobase of transcript per million mapped reads (FPKM) scores (Fig S2C) and pairwise scatterplot (Fig S2D) across QKI-ctrl and QKI-cKO MuSC samples. There were ∼400 DEGs and 600 AS events (ASEs) in purified MuSCs from QKI-ctrl and QKI-cKO mice (Fig 2B and Tables S1 and S2). Volcano plot analysis of DEGs revealed 167 down-regulated genes and 231 up-regulated genes (absolute log-twofold change > 1.2, false discovery rate (FDR) < 0.05, base mean > 20) (Fig 2C). Pathway enrichment analysis was performed on the QKI-mediated DEGs using the Enrichr software (Chen et al, 2013; Kuleshov et al, 2016) (Fig 2D). Genes that were down-regulated in QKI-cKO MuSCs were enriched for “muscle contraction” and “actin-myosin filament sliding” terms, which describe known functions of fully differentiated muscle (Fig 2E). Among the down-regulated genes were key structural components of muscle, including *Myl1*, *Myl4*, *Myh3*, *Mylph*, *Sspn*, and *Mybph*. The down-regulation of these targets was further validated with quantitative PCR (Fig 2F). The depletion of these muscle differentiation markers led us to investigate whether depletion of the QKI isoforms would lead to differentiation defects in cultured MuSCs. Therefore, we performed siRNA knockdown of QKI (siQKI) or Luciferase (siLuc) control in C2C12 murine myoblasts and grew the cells to confluency. C2C12 cells treated with siLuc and siQKI were capable of growing to confluency, indicating that cell viability was not affected. We then induced differentiation with reduced serum medium for 72 h (Fig 2G). We then calculated the fusion index as the percent of nuclei that were contained within differentiated myotubes. Control siLuc C2C12 cells had a fusion index of 77% ± 4.4%, and C2C12s treated with siQKI failed to differentiate with a fusion index of 1% ± 0.6% (Fig 2G and H). These findings corroborate with defects in the number of myogenic progenitors and muscle regeneration observed in QKI-cKO mice.

Table S1 Differentially expressed genes in QKI-cKO MuSCs.

Table S2 Skipped exons in QKI-cKO MuSCs.

Table S3 Primer sequences for RT–PCR and qPCR.

Differential AS events identified in QKI-cKO MuSCs were further categorized into skipped exon (SE, 68%), multiple skipped exon (MSE, 11%), retained intron (RI, 5%), alternative 3′ splice site (A3SS, 11%), and alternative 5′ splice site (A5SS, 4%) (Fig S2E). All significant SE events (Table S2) were visualized on integrative genomics viewer v2.8.13 (Robinson et al, 2011). Of the SE events, we observed 92 incidences of alternative last exon usage and 191 microexon splicing events (Fig S2F and Table S2). Our group has recently reported that QKI also regulates the splicing of microexons in microglia cells, and we have found 38 of these events were conserved in MuSCs (Lee et al, 2020) (Table S2). Furthermore, ∼65% of the AS exons in QKI-cKO MuSCs had QRE sequences in the neighbouring introns, and 45% of these QREs were located within 200 nucleotides of the exon, indicating that regulation of these AS events may be influenced directly by QKI. Overall, our transcriptomic analysis identified a dysregulated AS network in QKI-deficient primary MuSCs.

### The transcripts of asymmetric division components undergo dysregulated AS in QKI-deficient MuSCs

**Figure S3.**
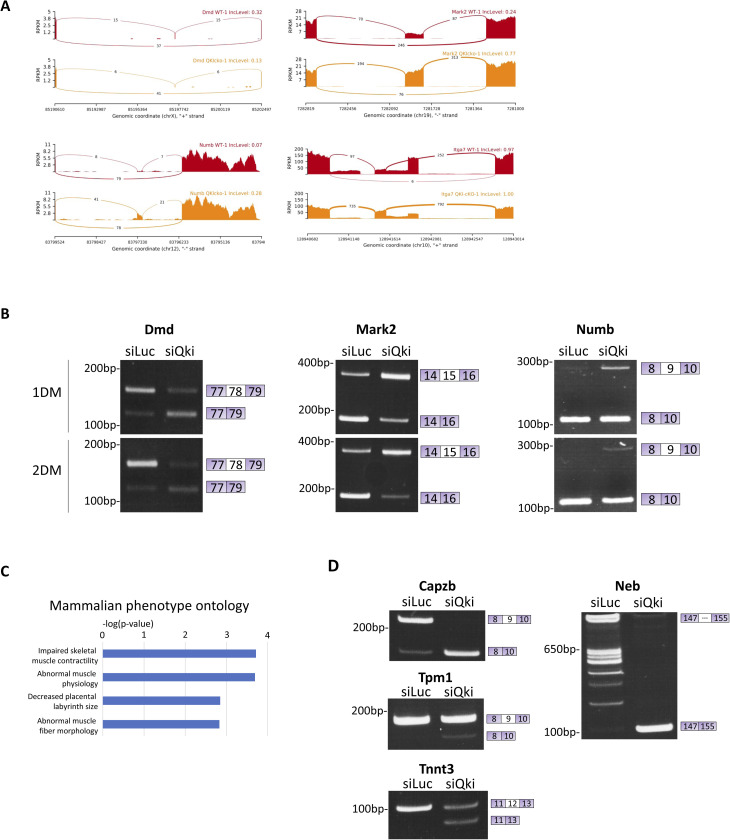
Components of MuSC polarity establishment undergo AS in QKI-deficient MuSCs. **(A)** Sashimi plots generated using rMATs software of asymmetric cell division factors. Dmd, Dystrophin; Mark2, Microtubule affinity regulating kinase; Itga7, Integrin alpha-7. WT: QKI-ctrl (red track); QKIcko: QKI-cKO (yellow track). **(B)** RT–PCR validation of SE events of *Dmd*, *Mark2*, and *Numb* in primary muscle stem cells (MuSCs) treated with siQKI or siLuc control and cultured in differentiation media for 1 d (1DM), or 2 d (2DM). **(C)** Pathway enrichment analysis carried out using Enrichr software of alternative last exon events in QKI-cKO versus QKI-ctrl MuSCs. **(D)** RT–PCR validation of alternative splicing events of select sarcomere components in proliferating MuSCs transfected with siQKI or siLuc. PCR products were separated on acrylamide gels and stained with ethidium bromide. The migration of DNA markers in base pairs (bp) is shown on the left, and alternative spliced exon and its inclusion/exclusion is depicted on the right. Capzb, Capping actin protein of muscle Z-line subunit beta; Tpm1, Tropomyosin 1; Tnnt3, Troponin T3; Neb, Nebulin.

Establishing polarity within the MuSC is a precursor to asymmetric cell division, and asymmetric cell division is a source of myogenic progenitor cells during muscle regeneration (Yin et al, 2013). Given the deficit in myogenic progenitors and severe differentiation and regeneration defects observed in QKI-deficient MuSCs, we further analyzed AS events in QKI-cKO MuSCs for components of cell polarity and asymmetric cell division. Interestingly, we observed AS patterns in *Dmd* (exon 78 exclusion), *Mark2* (exon 15 inclusion), and *Numb* (exon 9 inclusion) (Fig S3A). Notably, AS of *Dmd* exon 78 is known to occur in patients with myotonic dystrophy (DM1), resulting in expression of the embryonic form of dystrophin and leading to defects in mobility and muscle architecture (Rau et al, 2015).

**Figure 3.**
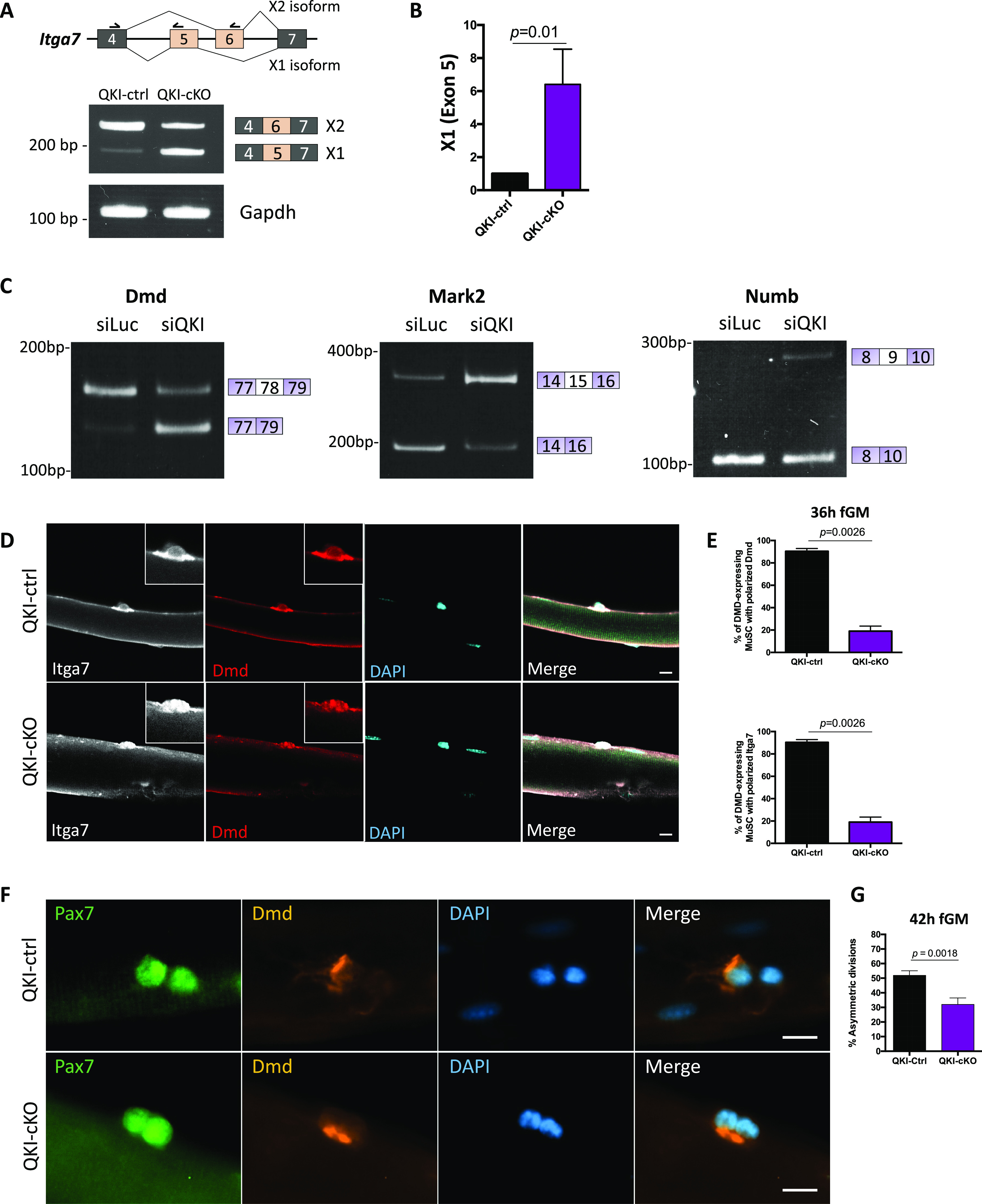
Components of muscle stem cell (MuSC) polarity establishment undergo alternative splicing (AS) in QKI-deficient MuSCs. **(A)** Upper panel: Diagram depicting splicing pattern of *Itga7* X1 and X2 isoforms. Arrows above exons 4, 5, and 6 represent primer direction. Lower panels: RT–PCR of *Itga7* X1 (exon 5) and X2 (exon 6) and *Gapdh* control in MuSCs isolated from QKI-ctrl and QKI-cKO mice. PCR products were separated on polyacrylamide gels and stained with ethidium bromide. The migration of DNA markers in base pairs (bp) is shown on the left, and exon inclusion/exclusion diagram is on the right. **(B)** Quantitative PCR for *Itga7* XI (exon 5) normalized to *Gapdh* control in MuSCs isolated from QKI-ctrl and QKI-cKO mice (n = 3 biological replicates, *P* = 0.01 unpaired *t* test). **(C)** RT–PCR validation of SE events of *Dmd*, *Mark2*, and *Numb* in proliferating MuSCs transfected with siLuc or siQKI. PCR products were electrophoresed on acrylamide gels and stained with ethidium bromide. The migration of DNA markers in base pairs (bp) is shown on the left, and inclusion/exclusion of the alternative spliced exon is depicted on the right. **(D)** Myofibers isolated from QKI-ctrl and QKI-cKO mice and cultured in fiber growth media for 36 h, stained with Itga7 (white) and Dmd (red), counterstained with DAPI (blue). Scale bar represents 10 μm. **(D, E)** Quantification of Dmd-expressing MuSCs from (D) that had polarized localization of Dmd (upper panel), and Itga7 (lower panel) (n = 3 mice per condition, 100 MuSCs quantified per mouse, unpaired *t* test, *P* = 0.0026). **(F)** Myofibers isolated from QKI-ctrl and QKI-cKO mice, cultured in fiber growth media for 42 h, stained with Pax7 (green) and Dmd (orange), and counterstained with DAPI (blue). Scale bars represent 10 μm. **(F, G)** Percentage of total divisions from (F) that were asymmetric based on inheritance of Dmd protein (n = 3 mice per condition, 100 MuSCs quantified per mouse, unpaired *t* test, *P* = 0.0018).

Interestingly, we also found alternative usage of exon 5 of the MuSC marker Integrin Alpha-7 (*Itga7*) in QKI-cKO (Fig S3A). The role of integrins in establishing cell polarity has been determined in intestinal cells (Goulas et al, 2012), but their function in MuSC polarity is not currently known. *Itga7* exons 5 and 6 are mutually exclusive and encode the extracellular linker domain which binds laminins in the extracellular matrix (ECM) of muscle (Collo et al, 1993; Song et al, 1993; Ziober et al, 1993). *Itga7* exon 5, but not exon 6, is present in the *Itga7-X1* isoform which efficiently binds laminin-511, one of the predominant laminin isoforms found in muscle ECM during embryogenesis and muscle regeneration. Whereas *Itga7-X2*, containing exon 6, but not exon 5, binds laminin-111, the other laminin isoform which is present during embryogenesis and muscle regeneration (von der Mark et al, 2002; Rayagiri et al, 2018). Laminin-211 is the predominant isoform in homeostatic adult muscle tissue and is recognized by both X1 and X2 isoforms (von der Mark et al, 2002). Interestingly, we observed a switch from the exon 6 isoform (X2) in QKI-ctrl MuSCs to the exon 5 isoform (X1) in QKI-cKO MuSCs (Fig S3A). To confirm the switch to the *Itga7-X1* isoform in QKI-cKO MuSCs, we performed RT–PCR on QKI-ctrl and QKI-cKO MuSCs using a common forward primer in exon 4, and reverse primers in exon 5 and exon 6, respectively. QKI-ctrl MuSCs expressed mainly the X2 isoform (exon 6) in MuSCs, whereas the QKI-cKO MuSCs expressed equivalent X1 (exon 5) and X2 isoforms (Fig 3A). We also confirmed the increase in *Itga7-X1* isoform in QKI-cKO MuSCs compared with QKI-ctrl by RT-qPCR of exon 5 (Fig 3B).

To confirm AS of the remaining MuSC polarity regulators *Dmd*, *Mark2*, and *Numb* in QKI-deficient MuSCs, we isolated primary MuSCs from C57BL/6 wild-type mice, cultured them in vitro, and transfected them with siLuc and siQKI for 2 d, followed by RNA isolation and RT–PCR. Primers were designed to flank the AS exon. The exclusion of *Dmd* exon 78 was observed in siQKI MuSCs compared with siLuc controls. Moreover, the inclusion of exons 9 and 15 for *Numb* and *Mark2* were confirmed (Fig 3C).

To determine whether QKI deficiency could also elicit these AS events in differentiating MuSCs, we transfected proliferating MuSCs with siLuc and siQKI for 2 d and then switched to reduced-serum differentiation medium for 24 and 48 h. We observed that the *Dmd*, *Mark2*, and *Numb* AS events were indeed conserved in differentiating MuSCs (Fig S3B).

Notably, the large proportion of AS transcripts which used an alternative last exon prompted us to perform pathway enrichment analysis of these targets, and we found enrichment for “striated muscle contraction,” and “impaired skeletal muscle contractility” terms (Fig S3C). Skeletal muscle contraction requires functional repeats of small subunits called sarcomeres, which are highly ordered structures consisting of hundreds of proteins including myosin and actin filaments (Rassier, 2017). QKI has been shown to regulate RNA metabolism in the contractile machinery of differentiated cardiomyocytes (Chen et al, 2021), and in the sarcomeres of zebrafish (Bonnet et al, 2017). RT–PCR analysis confirmed AS of the major sarcomere components *Capzb*, *Tnnt3*, *Tpm1*, and *Neb* in wild type primary MuSCs transfected with siQKI, but not siLuc (Fig S3D). Our findings suggest that the loss of QKI also leads to AS defects in major components of the skeletal muscle sarcomere.

### QKI deficiency leads to loss of polarization of Dmd and Itga7 proteins and a deficit in asymmetric MuSC divisions

**Figure S4.**
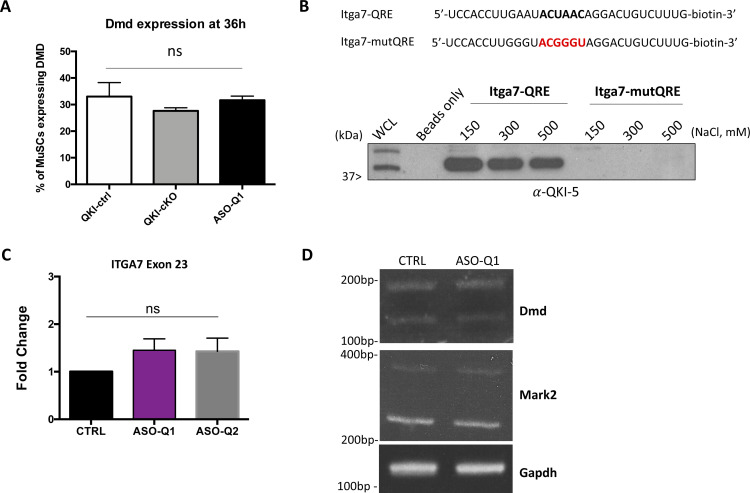
Inclusion of *Itga7-X1* (exon 5) is sufficient to induce loss of MuSC polarity and reduction of myogenic progenitors. **(A)** Quantification of proportion of Dmd-positive muscle stem cells (MuSCs) in QKI-ctrl, QKI-cKO, and ASO-Q1-transfected myofibers cultured for 36 h from Fig 4D (error bars represent mean ± SEM, n = 3 biological replicates per group. ns, not significant, unpaired *t* test). **(B)** Biotinylated RNAs were used for RNA pull-down assays. The mutated sequence disrupting the core of the QKI response element is indicated in red. Immunoblotting of QKI-5 after RNA pull-down assay using C2C12 total cell lysates. Increasing NaCl concentrations in the wash buffer was used to define the high affinity RNA binding of QKI-5. **(C)** RT-qPCR of exon 23 of *Itga7* in antisense oligonucleotide-treated MuSCs normalized to mock-transfected MuSCs (error bars represent mean ± SEM, n = 3 independent replicates, ns, not significant, unpaired *t* test). **(D)** RT–PCR validation of ASO-Q1 off-target alternative splicing events in proliferating MuSCs control (Ctrl) transfected or transfected with ASO-Q1. PCR products were separated on acrylamide gels and stained with ethidium bromide. The migration of DNA markers in base pairs (bp) is shown on the left.

To investigate whether polarization of Dmd and Itga7 protein would be affected by the AS defects observed in QKI-deficient MuSCs, we isolated myofibers from QKI-ctrl and QKI-cKO mice and performed immunofluorescence staining before the first cell division (which occurs at ∼40 h) by fixing the myofibers after precisely 36 h of culture. Itga7 has been shown to interact with dystrophin-associated glycoprotein complex, and thus is known to co-localize with Dmd in the MuSC (Dumont et al, 2015). The percentage of MuSCs that expressed Dmd was unchanged in QKI-cKO compared with QKI-ctrl control MuSCs (29.3% ± 6.7% in QKI-ctrl and 27.7% ± 1.2% in QKI-cKO) (Fig S4A). In QKI-ctrl MuSCs, both Dmd and Itga7 were localized to one side of the cell (i.e., polarized) in 91.7% of dystrophin-expressing MuSCs (Fig 3D and E). We observed a significant shift in QKI-cKO MuSCs, where Dmd and Itga7 were polarized in only 16.7% of dystrophin-expressing cells (Fig 3D and E). Therefore, loss of QKI results in polarization defects of Dmd and Itga7 in MuSCs.

The polarized Dmd protein in the MuSC is inherited by only one of the daughter cells following asymmetric division, whereas during symmetric divisions, Dmd is inherited by both daughter cells (Dumont et al, 2015). To determine whether the polarity defects observed in QKI-cKO MuSCs could contribute to reduced asymmetric divisions, we isolated myofibers from QKI-ctrl and QKI-cKO mice and performed immunofluorescence staining of Dmd just after the first cell division at 42 h (Fig 3F). Doublets of cells that had just completed division were analyzed for Dmd distribution, and divisions were qualified as asymmetric if Dmd was inherited by only one of the cells in the doublet. We did not account for apicobasal/planar orientation, as the apical daughter cell is often lost in ex vivo myofiber culture; therefore, the number of apicobasal versus planar divisions may be misrepresented. We observed a reduction in the percentage of asymmetric divisions in QKI-cKO compared with QKI-ctrl MuSCs (51.9% ± 10.0% in QKI-ctrl versus 32.1% ± 14.0% in QKI-cKO) (Fig 3G). Therefore, the polarity defects observed with QKI deficiency leads to a reduction in the number of asymmetric cell divisions.

### Inclusion of *Itga7-X1* (exon 5) is sufficient to induce loss of MuSC polarity and reduction of myogenic progenitors

**Figure 4.**
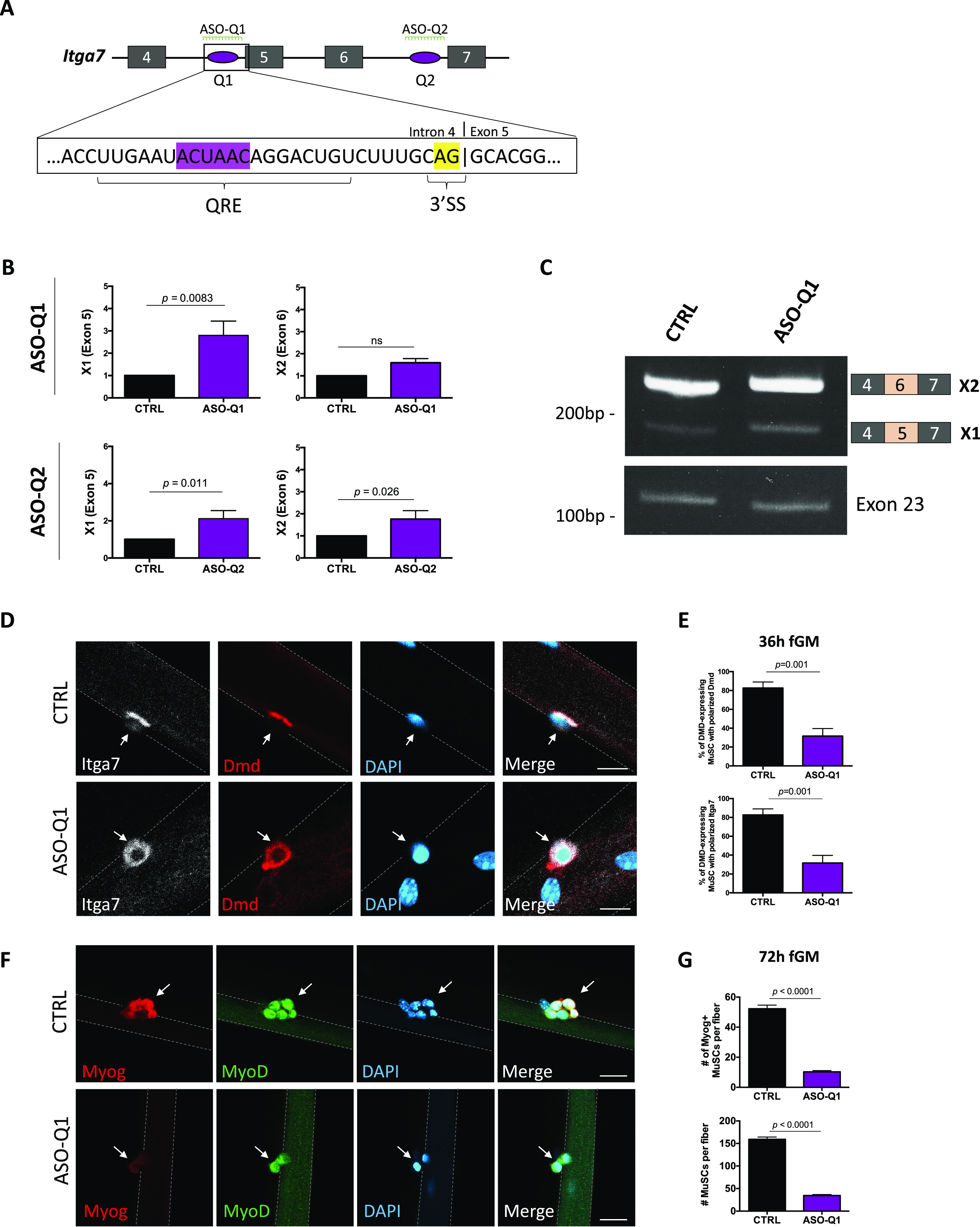
Inclusion of *Itg*a7-X1 (exon 5) is sufficient to induce loss of muscle stem cell (MuSC) polarity and reduction of myogenic progenitors. **(A)** Schematic of *Itga7* transcript from exon 4 to exon 7 is shown. QKI response elements represented as purple ovals; Q1 being most upstream and Q2 being downstream. The magnified box shows Q1 sequence with the core QKI response element site (ACUAAC) highlighted in purple, whereas the 3′ splice site (3′-SS) is highlighted in yellow. Location of antisense oligonucleotide (ASO) sequences against Q1 and Q1 (ASO-Q1 and ASO-Q2, respectively) are shown in green. **(B)** RT-qPCR for *Itga7* XI (exon 5) and *Itga7* X2 (exon 6), normalized to *Gapdh* in C57BL/6 wild type primary MuSCs treated with ASO against Q1 (ASO-Q1) and Q2 (ASO-Q2) (n = 3 replicates, ns, not significant, unpaired *t* test). **(C)** Semi-quantitative RT–PCR for *Itga7* XI (exon 5), *Itga7* X2 (exon 6), and *Itga7* exon 23 unchanged region control in C57BL/6 wild type primary MuSCs treated with ASO against Q1 (ASO-Q1). PCR products were separated on acrylamide gels and stained with ethidium bromide. The migration of DNA markers in base pairs (bp) is shown on the left, and exon inclusion/exclusion diagram is shown on the right. **(D)** Myofibers isolated from C57BL/6 wild type mice and transfected with ASO-Q1 4 and 16 h after adding culture media. Fibers were fixed 32 h after transfection and immunostained for Itga7 (white) or Dmd (Red), and counterstained with DAPI (blue). Scale bar represents 10 μm. **(E)** Quantification of Dmd-expressing cells with polarized Dmd (upper panel) and Itga7 (lower panel) (error bars represent mean ± SEM, n = 3 biological replicates, minimum 200 cells counted per group, *P* = 0.001, unpaired *t* test). **(F)** Myofibers isolated C57BL/6 wild type mice and transfected with ASO-Q1 or CTRL at 4 and 16 h after adding culture media. Fibers were fixed 72 h after addition of culture media, and immunostained for MyoD (green) and Myogenin (red) and counterstained with DAPI (blue). Scale bar represents 20 μm. White arrows point to MuSCs. **(G)** Quantification of Myogenin-positive MuSCs per myofiber (upper panel) and total number of MuSCs per myofiber (lower panel). Error bars represent mean ± SEM, n = 3 biological replicates, minimum 400 cells counted per group, *P* < 0.0001, unpaired *t* test.

To determine if the alternatively spliced components of asymmetric cell division could be direct targets of QKI-5, we interrogated the 200 nucleotide sequences flanking each of the AS exons of *Dmd*, *Mark2*, *Numb*, and *Itga7* for the QRE sequence. We found that only exon 5 of *Itga7* had a neighbouring QRE sequence (ACUAAY), which was located 14 nucleotides upstream of the 3′ splice site in intron 4 (Fig 4A). To confirm QKI-5 binds this QRE sequence, we generated a biotinylated RNA spanning the fragment of *Itga7* intron 4 which contains the QRE sequence (Itga7-QRE), and a biotinylated RNA harbouring a mutated QRE sequence (Itga7-mutQRE) (Fig S4B). The RNAs were bound to Streptavidin beads and RNA “pull-downs” were performed using total cell lysate from cultured C2C12 myoblasts. The bound proteins were washed with increasing NaCl concentration and eluted in sample buffer. The visualization of bound of QKI-5 was assessed by SDS–PAGE followed by immunoblotting. Indeed, the RNA containing the Itga7-QRE bound QKI-5 with high affinity as the interaction was maintained with 500 mM NaCl, whereas Itga7-mutQRE did not bind QKI-5 (Fig S4B). Therefore, QKI-5 binds to the intron 4 QRE sequence upstream of *Itga7* exon 5 to mediate AS at this location.

We next investigated whether the balance of X1/X2 *Itga7* isoforms could reproduce the polarity and myogenic progenitor defects we observed in QKI-cKO MuSCs. To re-create this defect, we opted for 2′-O-methyl ASOs targeting the *Itga7* intron 4 QRE sequence (ASO-Q1). We also identified a QRE sequence near the 3′ end of *Itga7* intron 6 and used this ASO as control (ASO-Q2) and given its distal location from exons 5 and 6, we expected this would not affect the splicing of exon 5 or 6. Wild type primary MuSCs were cultured in vitro and transfected with ASO-Q1 and ASO-Q2 either alone or in combination, or mock-transfected with water as a negative control. The cells were collected and RNA was isolated 2 d after transfection.

Quantitative PCR was performed to measure the relative inclusion of either exon 5 (X1) or exon 6 (X2) compared with an unspliced region of Itga7 (exon 23) among the ASO-treated MuSCs. Interestingly, we recapitulated the increased expression of the X1 isoform with transfection of ASO-Q1 (2.8 ± 0.37-fold increase normalized to *Gapdh*, *P* = 0.0083) (Fig 4B). Exon 23 was not differentially expressed with ASO treatment (Fig S4C). Unexpectedly, treatment with the control ASO-Q2 resulted in a slight increase in X1 expression, although to a lesser extent than ASO-Q1 (2.1 ± 0.25-fold increase normalized to *Gapdh*, *P* = 0.011). Expression of the X2 isoform (exon 6) was unaffected by treatment with ASO-Q1, and was slightly elevated by treatment with ASO-Q2 (1.76 ± 0.22-fold increase, *P *= 0.026) (Fig 4B). Semi-quantitative RT-qPCR was performed to visualize the increased expression of X1 (exon 5) with ASO-Q1 treatment, and to confirm unchanged expression in a common region within *Itga7* exon 23. Indeed, expression of exon 5 was increased in ASO-Q1-treated MuSCs compared with the mock-transfected with water control MuSCs. The common region in exon 23 remained unchanged, indicating that ASO treatment did not affect the overall expression level of *Itga7* (Fig 4C). The ASO-Q1 was specific for *Itga7*, as it did not influence *Dmd* and *Mark2* AS events (Fig S4D). These results indicate that masking the upstream QRE (i.e., Q1) with a targeted ASO was sufficient to promote *Itga7*-*X1* expression, albeit at a lower level than QKI-cKO MuSCs (compare Fig 4B and C with Fig 3A and B).

We next examined whether the ASO-Q1–mediated increased expression of *Itga7*-*X1* containing the extracellular linker region could reproduce the polarity defects observed in QKI-cKO MuSCs. Myofibers were isolated from C57BL/6 wild type mice and transfected with ASO-Q1 or mock transfected with water as a negative control (CTRL). Transfection was performed 4 and 16 h after addition of fiber growth medium (fGM), and myofibers were fixed 36 h after the addition of fGM. Co-immunostaining of Itga7 and Dmd revealed a significant reduction of polarization of both proteins in ASO-Q1 transfected myofibers (28.4% ± 1.8%) compared with CTRL cultures (83.0% ± 4.3%) (Fig 4D and E).

ASO-Q1 treatment of myofibers was then extended to 72 h of culture to assess the production of Myog^+^ myogenic progenitors. Myofibers were transfected twice with ASO-Q1 or water CTRL at 4 and 16 h after adding fGM and fixed after 72 h in culture. We observed a striking reduction in Myog^+^ myogenic progenitors per myofiber in ASO-Q1 treated myofibers (10.4 ± 2.8) versus CTRL cultures (52.3 ± 7.6), and a reduction in the total number of MuSCs per myofiber (159.3 ± 15.7 in CTRL versus 35.1 ± 7.4 in ASO-Q1) (Fig 4F and G). These data show that inducing the expression of *Itga7-X1* isoform by targeting the intron 4 QRE re-creates the MuSC polarity and myogenic progenitor defects observed in QKI-cKO MuSCs.

## Discussion

In the present study, we define a role for QKI-5 and Itga7 X1/X2 isoforms in MuSC polarity. We report that major polarity-determining factors including *Itga7* and *Dmd* undergo defective AS in QKI-deficient primary MuSCs. QKI-deficient MuSCs exhibited a loss of cell polarity and loss of the myogenic progenitor population. Treatment of wild type MuSCs with ASOs directed against the QRE in *Itga7* intron 4 led to exon 5 inclusion and expression of the *Itga7-X1* isoform known to express extracellular linker domain and interfere with polarization of both Dmd and Itga7. Our findings identify QKI-5 as a critical AS regulator of polarity in MuSCs.

The loss of cell polarity in QKI-deficient MuSCs suggests that one possible mechanism through which the pool of myogenic progenitors is depleted is through a defect in asymmetric cell division. Asymmetric cell division is essential for diversification of cell types during development and specification of stem cells in adults (Knoblich, 2010; Fuchs & Blau, 2020). In Duchenne muscular dystrophy (DMD), the ability of MuSCs to divide asymmetrically is impeded by loss of the dystrophin protein, which plays a role in establishing the cell polarity that is required for asymmetric division (Dumont et al, 2015). The deficiency in myogenic progenitors arising from Dmd-deficient MuSCs is accompanied by hyperplasia of non-committed stem cells in Kottlors & Kirschner (2010), Dumont et al (2015), and Chang et al (2016). The defect in cell polarization (Fig 3D) and lack of myogenic progenitors formed by QKI-cKO MuSCs (Fig 1C) presents as a phenocopy of Dmd-deficient cells. Notably, however, we did not observe stem cell hyperplasia in QKI-cKO mice, but rather a reduction in the total number of MuSCs (Fig 1D), suggesting that the QKI-cKO phenotype may be only partly mediated by defects in AS of Dmd.

The MuSC surface marker Itga7 has been shown to affect the generation of myogenic progenitor cells and muscle differentiation (Mayer et al, 1997; Rooney et al, 2009; Ding et al, 2020). The expression of *Itga7-X1* isoform in QKI-deficient MuSCs and subsequent loss of MuSC polarity provides evidence that maintaining a high *Itga7-X2*/*Itga7-X1* isoform ratio is a requirement for MuSC polarity.

The *Itga7-X1* (exon 5) and *Itga7-X2* (exon 6) isoforms are expressed in a mutually exclusive manner and are regulated during development. The X1 isoform is observed in embryogenesis during the development of skeletal muscle, whereas the X2 isoform in found in adult skeletal muscle (Collo et al, 1993; Song et al, 1993; Ziober et al, 1993; von der Mark et al, 2002). Although the X1 isoform is needed during embryonic skeletal muscle development, our findings suggest that it is “toxic” or not tolerated during the regeneration of adult muscle tissue. It is known that the Itga7 X1 and X2 isoforms have varied binding affinities to different laminin isoforms in the muscle ECM. The X1 isoform of *Itga7* preferentially binds laminin-511, whereas the X2 isoform binds laminin-111. The X1 isoform can also bind laminin-111 but with much lower affinity (von der Mark et al, 2002). Therefore, our findings lead us to speculate that the X2/X1 ratio in QKI-cKO or ASO-Q1 treated MuSCs may be detrimental to MuSC polarity in part due to incompatible interactions with the changing landscape of extracellular laminin isoforms in adults.

The skipping of *Itga7* exon 5 in QKI-ctrl MuSCs suggests that binding of QKI-5 to its QRE within intron 4 may sterically prevent access of the U2 snRNP splicing machinery to the 3′ splice site, whereas in QKI-cKO MuSCs the absence of QKI promotes splicing and inclusion of exon 5 rather than exon 6. Treatment of wildtype MuSCs with an ASO that binds to the intron 4 QRE (ASO-Q1) results in exon 5 inclusion. One possible mechanism through which ASO-Q1 increases exon 5 inclusion is through binding and masking a splicing silencer element (SSE). The use of ASOs to restore exon expression through this mechanism has been demonstrated previously. For example, an ASO-walking technique was used to identify an SSE in the *IKBKAP* pre-mRNA responsible for the pathological skipping of exon 20 in familial dysautonomia (Sinha et al, 2018).

RBPs such as QKI and their effects on AS play an important role in disease (Lukong et al, 2008; Scotti & Swanson, 2016). For example, disruption of the *QKI* gene in a human patient resulting in haploinsufficiency led to several clinical manifestations, including severely reduced muscle tone (i.e., hypotonia) (Backx et al, 2010). Our findings provide further evidence of the important contributions of RBPs and RNA metabolism in muscle wasting disease. Several splicing aberrations are identified in muscle wasting disease (Nakka et al, 2018). For example, aberrant splicing in Myotonic dystrophy type I (DM1) is mediated through sequestration of the Muscleblind-like RBP to pathological CUG repeats within the DM protein kinase (*DMPK1*) RNA (Philips et al, 1998; Charlet et al, 2002; Thomas et al, 2017). Subsequent loss of Muscleblind-like function leads to AS of several muscle-specific targets, including the chloride channel *CLCN1*, wherein a premature stop codon is induced leading to hyperexcitability of myofibers (Charlet et al, 2002; Mankodi et al, 2002). Development of strategies to target such pathological splicing events include the use of ASOs. Using ASO to specifically target the 3′ splice site of *CLCN1* exon 7a, or the more general CUG repeat region of *DMPK1*, improves myopathy in DM1 mouse models (Wheeler et al., 2007, 2009). Given the recapitulation of muscle disease phenotypes that we see with a single AS event of *Itga7* exon 5, we can envision the use of ASOs to target similar AS aberrations in components of asymmetric cell machinery. Indeed, splicing aberrations in *Itga7* which modulate its function are reported to be involved in myopathy (Hayashi et al, 1998; Pegoraro et al, 2002).

In summary, we report QKI-5 as a major regulator of splicing in skeletal MuSCs and as being essential for maintaining the splicing isoform pattern that drives MuSC polarity and production of myogenic progenitors. QKI-deficient mice had severe muscle regeneration defects arising from a lack of these progenitor cells. AS of *Itga7* mediated by a QRE-targeted ASO, or in QKI-deficient MuSCs, led to cell polarity defects thus identifying a role for *Itga7* isoforms in establishing cell polarity.

## Materials and Methods

### Mice

C57BL/6 (000664; Jackson Laboratory) were the wild type mice used for MuSC FACS purification and myofiber isolation where indicated. QKI-cKO mice are maintained in a C57BL/6 background with two loxP sites flanking exon two of the *QKI* gene (QKI^2lox/2lox^), and were generated previously (Darbelli & Richard, 2016). QKI^2lox/2lox^ (QKI-ctrl) mice were crossed with C57BL/6 mice expressing CreERT2 recombinase under the *Pax7* promoter (#017763; Jackson Laboratory) to generate QKI-cKO mice with 4-hydroxytamoxifen-induced QKI deficiency specifically in MuSCs. To induce Cre recombinase, 4-hydroxytamoxifen (TAM, T5648; MilliporeSigma) was dissolved in corn oil (C8267; MilliporeSigma) to 1 mg/ml, and 100 μl was injected intraperitoneally in 6- to 8-wk-old mice once daily for five consecutive days. Experiments were conducted 10 d following the final injection of 4-hydroxytamoxifen. Sex- and age-matched mixed populations of males and females were used for each genotype for all experiments. All mouse husbandry and experiments were conducted in accordance with the Institutional Animal Care and Use Committee (IACUC) of McGill University. All animal procedures conducted at McGill were approved by the Animal Welfare Committee of McGill University (protocol #3506).

### Myofiber isolation and culture

Wild-type C57BL/6, QKI-ctrl, and/or QKI-cKO mice were euthanized and their extensor digitorum longus muscles were dissected using standard dissection techniques. Isolated muscles were incubated with 0.4% collagenase (c0103; Sigma-Aldrich) in DMEM for 30 min at 37°C and 5% CO_2_. Whole muscle was then triturated with a plastic disposable Bohr pipette to dissociate individual fibers from the whole muscle as described previously (Gallot et al, 2016). To mimic activating conditions, fibers were cultured in fGM (DMEM plus 20% FBS, 1% chick embryo extract [100-163P; Gemini Bio], 2.5 ng/ml bFGF [13256-029; Gibco], and 1% penicillin/streptomycin) at 37°C and 5% CO_2_. For quiescent satellite cell analysis, fibers were fixed immediately after dissociation using 2% PFA prepared fresh in 1× PBS.

### Myofiber immunofluorescence

Cultured myofibers were fixed in 2% PFA at the indicated time points and washed twice with 1× PBS. Fixed myofibers were then permeabilized with 0.2% Triton X-100, 0.125 M glycine in 1× PBS for 15 min at RT. Blocking followed for 1 h at RT with 2% BSA, 5% horse serum and 0.1% Triton X-100. Primary antibodies were diluted in blocking buffer to detect Pax7 (1:100; Developmental Studies Hybridoma Bank [DSHB]), MyoD (sc-304; Santa Cruz Biotechnology), Myogenin (F5D; DSHB), Dystrophin (MANDRA1[7A10]; DSHB), QKI-5 (AB9904, AB9906, respectively; MilliporeSigma). After 16-h incubation at 4°C, fibers were washed three times with 1× PBS for 10 min. Secondary antibody (AlexaFluor anti-mouse or anti-rabbit 488 or 568 nm), or AlexaFluor-647–conjugated primary antibody against Itga7 (FAB3518R; R & D Systems) was used at a dilution of 1:400 in blocking buffer for 1 h in the dark at RT. Myofibers were washed three times for 10 min with 1× PBS. Finally, the myofibers were transferred to a microscope slide outlined using an ImmEdge hydrophobic barrier pen (H-4000; Vector Laboratories) and mounted with ProLong Gold Antifade Mountant with DAPI (P36935; Thermo Fisher Scientific). Fiber-associated MuSCs were then visualized on a Zeiss Axio Imager M1 microscope (Carl Zeiss) or LSM800 Airyscan confocal microscope and resulting images were analyzed using Zeiss’ ZEN Digital imaging suite software.

### Primary MuSC isolation

Skeletal muscle tissue was isolated from the abdominal and diaphragm muscles of wild type C57BL/6, QKI-ctrl, and/or QKI-cKO mice and MuSCs were isolated as previously described using FACS (Pasut et al, 2012). Briefly, dissected muscles were minced with dissection scissors and digested with collagenase/dispase solution (2.4 U/ml collagenase D, 2.4 U/ml Dispase II in Ham’s F10 media) at 37°C for 1 h. Digested tissue was triturated and filtered through a 40-μM cell strainer. Cells were pelleted for 18 min at 300*g* and resuspended in 1% BSA/PBS. Cells were stained for 15 min RT with Itga7-AlexaFluor-647 (FAB3518R; R & D Systems) for positive selection, and PE-CD45 (Invitrogen), PE-CD11b (PE-CD31; Invitrogen [BD Pharmigen], and PE-ScaI [BD Pharmigen]) for negative selection. Hoescht was used to gate living cells. Cells were washed once with 1% BSA/PBS and pelleted before final resuspension and one last filter through a 40 μM cell strainer. ITGA7+/CD45−/CDCD11b−/Sca1−/Hoechst+ Cells were sorted into full myoblast growth media (GM) using the FACSAriaIII cell sorter (BD Biosciences).

### MuSC growth and differentiation culture

Purified MuSCs were seeded onto collagen-coated plates and expanded in GM (Ham’s F10 media with 20% FBS, 12.5 ng/ml human recombinant bFGF [13256-029; Gibco], and 1% penicillin/streptomycin) at 37°C and 5% CO_2_. The medium was replenished every 2 d. To differentiate MuSCs into myotubes, MuSCs were grown to 90% confluency in GM, washed twice with 1× PBS, and switched to differentiation medium (DM: DMEM, 1% FBS, and 1% Penicillin/Streptomycin). C2C12 myoblasts were grown in DMEM, 10% FBS, and 1% Penicillin/Streptomycin at 37°C and 5% CO_2_. To induce differentiation, C2C12 myoblasts were grown to 90% confluency and cultured in DMEM, 1% FBS, and 1% Penicillin/Streptomycin at 37°C and 5% CO_2_.

### In vivo muscle regeneration and cross-sectional immunofluorescence

The right TA muscles of QKI-ctrl or QKI-cKO mice were injected once with 50 μl of 10 μM CTX (217502; Sigma-Aldrich) to induce muscle injury. After 3 wk, mice were euthanized, and the injured and contralateral control TA were dissected using standard dissection techniques. Dissected TA muscles were fixed in 0.5% PFA for 2 h at 4°C, and subsequently equilibrated in 20% sucrose in 1× PBS overnight at 4°C. The following day, TA muscles were rapidly frozen in liquid nitrogen-cooled isopentane and embedded in OCT compound (23-730-571; Thermo Fisher Scientific). Frozen TA muscles were cut into 10 μM sections using a Leitz cryostat directly onto Fisher Superfrost Plus microscope slides (12-550-150) for immunofluorescence staining. Resulting tissue sections were permeabilized for 12 min with 0.2% Triton X-100, 0.125 M glycine in PBS at RT. Blocking followed with M.O.M blocking reagent (MKB-2213-1; Vector Laboratories) for 1 h at RT. Primary antibodies were diluted in blocking reagent to detect Pax7 (Pax7; DSHB) and laminin (L9393; Sigma-Aldrich). After 16-h incubation at 4°C, sections were washed three times with 1× PBS for 10 min. Secondary antibody (AlexaFluor anti-mouse or anti-rabbit 488 or 568 nm) was used at a dilution of 1:400 in blocking reagent for 45 min in the dark at RT. Sections were then washed three times for 10 min with 1× PBS. Tissue sections were mounted with ProLong Gold Antifade Mountant with DAPI (P36935; Thermo Fisher Scientific) and covered with a coverslip. Visualization was performed on a Zeiss Axio Imager M1 microscope (Carl Zeiss), and resulting images were analyzed using Zeiss’ ZEN Digital imaging suite software.

### RNA-seq sample preparation and analysis

Primary MuSCs were purified from 6- to 8-wk-old QKI-ctrl or QKI-cKO mice (n = 3 biological replicates) using FACS gating of ITGA7+/CD45−/CDCD11b−/Sca1−/Hoechst+ cells. Purified cells were expanded ex vivo in myoblast growth medium for 72 h, pelleted, and bulk RNA was isolated using the PicoPure RNA extraction kit according to the manufacturer’s protocol (4346906; Applied Biosystems). RNA quality was assessed using an Agilent Tapestation 4200. RNA sequencing libraries were generated with TruSeq stranded mRNA Sample Prep Kit with TruSeq Unique Dual Indexes (Ilumina). The resulting libraries were multiplexed and sequenced with 100 bp paired-end reads on the Illumina NovaSeq platform. Samples were subsequently demultiplexed with bcl2fastq v2.20 Conversion Software from Illumina. Illumina adaptor sequences were then removed using Trimmomatic v0.39 software. Trimmed sequences were subsequently aligned to the mm10/GRCm38 genome using STAR v2.7. Mapped reads were then processed using the Cufflinks software suite. FPKMs and fragment counts were scaled using the geometric means of fragment counts across all libraries. Significant changes in transcript expression were classified as having a log fold change greater than 2, base means larger than 20, and FDR less than 0.05. Expression plots were generated using CummeRbund. AS events in QKI-cKO MuSCs were quantified using rMATS v4.0.2 and Gencode vM23 gene annotations using untrimmed paired-end reads. Events with an FDR of less than 0.05 were accepted as significant. Pathway enrichment analysis of DEGs and AS events was performed using Enrichr.

### RNA isolation, cDNA synthesis, qPCR, RT–PCR, and polyacrylamide gel electrophoresis

TRIzol (Invitrogen) was used to isolate RNA from cultured primary MuSCs per the manufacturer’s instructions. 1 μg of total RNA was used for cDNA synthesis using M-MLV reverse transcriptase (M1701; Promega), followed by qPCR analysis with targeted primers using PowerUp SYBR Mastermix (A25777; Life Technologies) run on QuantStudio7 real-time PCR system (Thermo Fisher Scientific). mRNA was quantified with the ΔΔCt method, normalizing to Gapdh and/or Hprt and 18s mRNA levels as indicated. Splicing assays were performed using cDNA isolated as described above and specific primers which flanked the indicated exon for PCR. Resulting PCR products were separated on a TBE-based polyacrylamide gel and DNA was stained with ethidium bromide. All PCR reactions were repeated in triplicate. A complete list of RT-PCR and qPCR primers is provided in Table S3.

### Western blotting

Proteins from total cell lysate (150 mM NaCl, 50 mM Hepes pH 7.4, and 1% Triton X-100) were separated by SDS–PAGE and transferred onto nitrocellulose membranes using the Trans-Blot turbo transfer system (Bio-Rad). Membranes were stained with Ponceau Red to confirm equal loading, and then blotted with the primary antibodies against myosin heavy chain (MF-20; DSHB), QKI-5 (AB9904, AB9906, respectively; MilliporeSigma) overnight at 4°C. After three washes in TBST, membranes were incubated with HRP-conjugated secondary antibodies (Sigma-Aldrich) for 45 min and visualized on X-ray films with Western Lightning Plus ECL (PerkinElmer).

### Transfection of siRNA and ASOs in primary MuSCs

FACS-isolated primary MuSCs were transfected with siRNA designed to target QKI (5′-GGACUUACAGCCAAACAAC-3′) and luciferase as a negative control (5′-CGUACGCGGAAUACUUGA-3′). siRNA transfections were carried out using Lipofectamine RNAiMAX reagent (13778075; Thermo Fisher Scientific) per manufacturer’s instructions. Cells were harvested 48 h after transfection for total RNA isolation. 2′-O-methyl ASOs were designed to target the exon 5 upstream QRE (ASO-Q1: 5′-GUCCUGUUAGUAUUCAAGGUGG-3′), and the downstream QRE (ASO-Q2: 5′-CGAUUACUGUGAGUGAUUAUCCAAC-3′) to induce exon 5 inclusion. For cultured primary MuSCs, 50 nM of each ASO or water control were transfected using the Lipofectamine 3000 transfection reagent (L3000001; Thermo Fisher Scientific) according to the manufacturer’s instructions. Cells were harvested 48 h after transfection for total RNA isolation. For myofibers, a first transfection was performed with 50 nM ASOs 4 h after addition of fGM. A second transfection was performed 12 h after the initial transfection. Myofibers were subsequently fixed 36 or 72 h after addition of culture medium as indicated.

### RNA binding assay

Streptavidin Mag Sepharose magnetic beads (28985738; Cytiva Life Sciences) were incubated with 1 µg of biotinylated RNA (IDT) for 30 min at 4°C with end-over-end mixing.

C2C12 myoblast lysate (150 mM NaCl, 50 mM Hepes pH 7.4, 1% Triton X-100, supplemented with 40 U/ml RNase inhibitor and protease inhibitors) was then added to the RNA-Streptavidin mixture and incubated at 4°C with end-over-end mixing for 1 h. The beads were then washed three times with lysis buffer containing increasing salt concentrations (150, 300, and 500 mM NaCl). Protein samples were separated with SDS–PAGE and immunoblotted for QKI-5.

## Data Availability

The RNA-seq data from this publication have been deposited to Gene Expression Omnibus under the accession number GSE193899.

## Supplementary Material

Reviewer comments
